# Do item-writing flaws reduce examinations psychometric quality?

**DOI:** 10.1186/s13104-016-2202-4

**Published:** 2016-08-11

**Authors:** João Pais, Artur Silva, Bruno Guimarães, Ana Povo, Elisabete Coelho, Fernanda Silva-Pereira, Isabel Lourinho, Maria Amélia Ferreira, Milton Severo

**Affiliations:** 1 Departamento de Educação e Simulação Médica, Piso 6, Faculdade de Medicina da, Universidade do Porto, Alameda Prof. Hernâni Monteiro, 4200-319 Porto, Portugal; 2 Departamento de Epidemiologia Clínica, Medicina Preditiva e Saúde Pública, Faculdade de Medicina da, Universidade do Porto, Alameda Prof. Hernâni Monteiro, 4200-319 Porto, Portugal; 3 Departamento de Anatomia, Faculdade de Medicina da, Universidade do Porto, Alameda Prof. Hernâni Monteiro, 4200-319 Porto, Portugal

**Keywords:** Assessment, Examination, Item-writing flaws, Multiple-choice questions, Clinical anatomy, Psychometric characteristics

## Abstract

**Background:**

The psychometric characteristics of multiple-choice questions (MCQ) changed when taking into account their anatomical sites and the presence of item-writing flaws (IWF). The aim is to understand the impact of the anatomical sites and the presence of IWF in the psychometric qualities of the MCQ.

**Results:**

800 Clinical Anatomy MCQ from eight examinations were classified as standard or flawed items and according to one of the eight anatomical sites. An item was classified as flawed if it violated at least one of the principles of item writing. The difficulty and discrimination indices of each item were obtained. 55.8 % of the MCQ were flawed items. The anatomical site of the items explained 6.2 and 3.2 % of the difficulty and discrimination parameters and the IWF explained 2.8 and 0.8 %, respectively.

**Conclusions:**

The impact of the IWF was heterogeneous, the *Writing the Stem* and *Writing the Choices* categories had a negative impact (higher difficulty and lower discrimination) while the other categories did not have any impact. The anatomical site effect was higher than IWF effect in the psychometric characteristics of the examination. When constructing MCQ, the focus should be in the topic/area of the items and only after in the presence of IWF.

**Electronic supplementary material:**

The online version of this article (doi:10.1186/s13104-016-2202-4) contains supplementary material, which is available to authorized users.

## Background

The Clinical Anatomy course makes a breakthrough in students’ anatomical education by focusing on the capability to apply anatomical knowledge with medical reasoning to solve clinical problems [1, 2]. One of the objectives was to use the basic anatomical knowledge in the interpretation of the patients’ symptoms and their macroscopic morphological alterations.

The most common methods to assess anatomy knowledge are: multiple choice questions (MCQs), extended matching questions (EMQs), short essay questions (SEQ), and identifying tagged structures (spotters) in specimens (practical examination).

The rules of writing good MCQ are well documented in the research literature [3, 4]. Haladyna et al. summarized these rules in a taxonomy of 31 items-writing guidelines to help in the construction of MCQ. These guidelines are divided into five main categories: *Content Concerns, Formatting Concerns, Style Concerns, Writing the Stem* and *Writing the Choices.*

The effect of item-writing rules on examination psychometric indices, such as item difficulty and discrimination has been studied [3]. However, most studies evaluated the effect of single-item flaw or the 31-items flaws on the psychometric characteristics of items; these studies did not assess the effect by the main five categories of item-writing flaws (IWF).

For example, the use of the negative form in the stem of MCQ was studied by Tamir [5]. In his work, Tamir concluded that items which required higher cognitive skills were more difficult when the negative form was used. When the students were asked to justify their choices they tended to follow a more complex thought process when the MCQ was in the negative form. Another example is the effect of using the option “none-of-the-above” in the psychometric indices discussed in a study made by Rich et al. [6]. In this work it was recommended that this option was used cautiously. When comparing MCQ containing the option “none-of-the-above” with conventional items without this option, Rich et al. found that there was a decrease of the difficulty index (the item was more difficult to answer correctly) and a decrease in the discrimination index. The recommendation of the authors regarding the “none-of-the-above” option was against its use when other good distracters could be created.

The effect of at least one item-flaw compared with none was studied by Tarrant et al. Their work consisted of the analysis of the impact of the IWF in MCQ in the students’ achievement [7, 8]. Although there were no significant statistical differences regarding the difficulty of the items, Tarrant et al. concluded that the presence of IWF had a negative impact on the performance of high-achieving students, giving an advantage to borderline students that likely relied on test-wiseness. In another study, conducted by Downing [9], it was concluded that the presence of IWF could lead to a misclassification of students as failed when they should be classified as passed, with a percentage that could go as high as 10–15 % of all tested students, proving the negative impact that IWF can have in students’ performance. These IWF’s will make the items more difficult for some students adding construct-irrelevant variance to the score and threaten the test validity [10]. The IWF’s will add some unintended construct not directly linked to our primary construct of interest.

The presence of the IWF is not the only factor that causes changes in the examination psychometric. In a previous study [11] it was observed that two-thirds of the problematic items were concentrated in specific anatomical regions of the examination. This study showed that the anatomical regions of the items were associated with the difficulty and discrimination index of MCQ. There are several reasons that can explain this finding, the first is that the item construction in the assessment was not similar between regions, the second reason is the quality of teaching (materials, time, etc.) also was not similar or even the cognitive load of anatomy content vary from region to region. In a previous study students point out that anatomy being taught by region was shortcoming [12].

The aims of the study were to evaluate IWF prevalence vary according to the anatomical region and if this is the main explanation for the effect of the anatomical region on the psychometric indices, and finally assess the effect of the five main categories of the IWF on the psychometric indices.

## Methods

The research design was cross-sectional and observational. The participants were year 2 medical students that fulfil in the end-of-year high-stakes Clinical Anatomy examination between 2008 and 2011.

Clinical anatomy was integrated in the second year of the medical curriculum of the Faculty of Medicine of University of Porto (FMUP).

Clinical anatomy could be divided into different topics, each one with its specific anatomical site. There were eight different topics, seven of them referring to the anatomical sites of the human body (Head, Neck, Thorax, Abdomen, Pelvis and Perineum, Upper Limb, Lower Limb) and one that focused on the different imaging methods to study the human body (Imagiology). Table 1 describes the median number of hours lectured about each one of the eight topics of Clinical Anatomy along the course and the distribution of MCQ by topics.

**Table 1:** Descriptive statistics of distribution of class hours and number of items by examination by content area

Content	Median of number of hours lectured (range)	%^a^	Median of the number of questions (range)	%^a^
Abdomen	5.3	23.8	18 (17–20)	18.0
Pelvis and perineum	5.0	21.7	18 (17–19)	18.0
Upper limb	1.5	6.5	9 (8–10)	9.0
Lower limb	1.5	6.5	9 (8–9)	9.0
Neck	2.3	9.8	15 (12–19)	15.0
Thorax	4.3	18.5	18 (15–19)	18.0
Head	2.3	9.8	11 (6–14)	11.0
Imagiology	1.0	4.3	3 (0–4)	3.0

^a^Calculated using the median value

### Definition of the classification of the items

Eight hundred standard MCQ (five different response options in which only one is the right answer) were analyzed. These items were taken from Clinical Anatomy examinations from 2008 to 2011. In each year there were two final examinations which comprise a total of eight examinations.

The MCQ were classified according to the anatomical site/topic and Haladyna’s taxonomy [3]. The anatomical sites/topics were: abdomen, pelvis and perineum, upper limb, Lower Limb, Neck, Thorax, Head and Imagiology.

According to the taxonomy the items were classified as a standard or a flawed item. An example of a flawed MCQ with the option “none-of-the-above”.

A patient with a left kidney abscess (pus accumulation) can show during the evolution of the disease another abscess in the left groin. What is the anatomical explanation for this evolution) (select the CORRECT answer):

- Inferior closure of the renal fascia
- Hematogenic dissemination of the infection
- Proximity of the kidney and the descendent colon
- Invasion of iliopsoas sheet
- None of the above

**Fig. 1:**
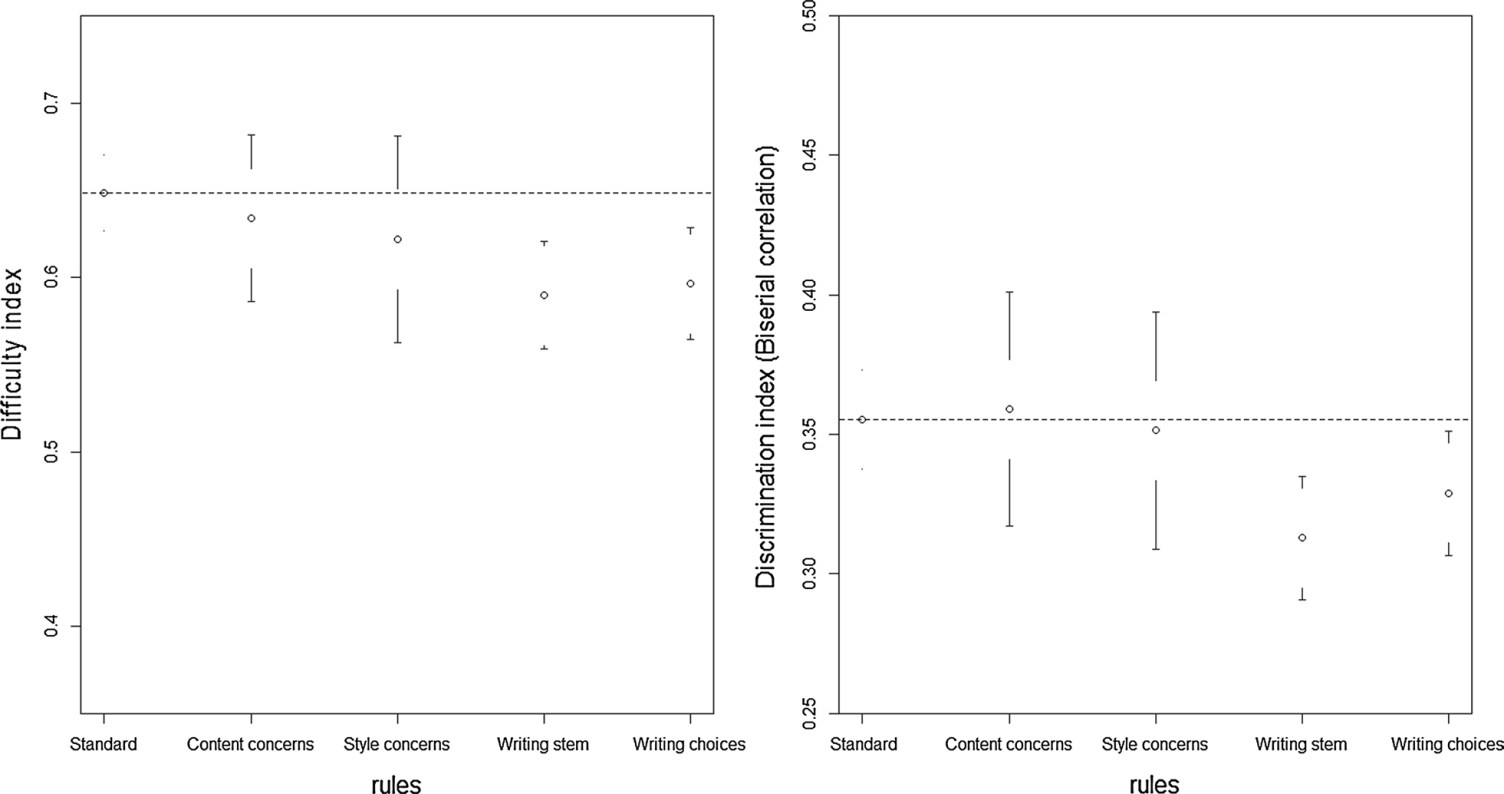
Difficulty and discrimination items indices by presence or absence of item writing flaw. The IWF were grouped according to the areas of flaw

An item was considered flawed if it violated at least one of the guidelines presented in the taxonomy developed by Haladyna et al. [3]. A standard item was one that did not violate any of the same guidelines. We also used the taxonomy to classify the items in terms of flaw areas: *Content Concerns* (for example, “rule 1: single content and behavior”), *Formatting Concerns* (for example, “rule 9: format vertically”), *Style Concerns* (for example, “rule 13: minimize reading”), *Writing the Stem* (for example, “rule 17: use positive, no negatives”) and *Writing the Choices* (for example, “rule 24: choice length equal”). The category *Formatting Concerns* did not appear in the analysis because none of the items contained a flaw of this group (Fig. 1).

The classification was made by four raters (2 teachers and 2 students), blinded to all item performance data and independently from each other.

The agreement between raters about the anatomical site and taxonomy previous to the consensus process was excellent (Fleiss kappa: 0.89) and fair (Fleiss kappa: 0.3), respectively [6].

### Definition of item characteristics

Difficulty and discrimination indexes were computed for each item. The item difficulty was estimated by the proportion of students answering the item correctly and the item discrimination was estimated by biserial correlation between the item and the total score. We considered an item with difficulty index lower than 0.3 as hard, between 0.3 and 0.8 as medium and higher that 0.8 as easy [13]. We considered discrimination lower than 0.2 as weak, between 0.2 and 0.3 as fair, between 0.3 and 0.4 as good and higher 0.4 as very good [14].

### Statistical analysis

The association between the item-writing flaws and the anatomical site was obtained by using the Chi square test. ANOVA and Kruskal–Wallis tests were used to compare the difficulty and discrimination indices by anatomical site and by the presence or absence of item-writing flaws.

The mean and the respective confidence interval for the difficulty and discrimination indices by type of writing flaws and content area were estimated using the Normal distribution approximation.

A random effect model with three random effects (the presence of item writing flaws, anatomical site and the examination) was used to estimate the variance percentage of the difficulty and discrimination indices explained by the presence of item writing flaws and by the anatomical site in order to have a general measure of the effect of each factor.

## Results

Table 2 shows the description of eight examinations.

**Table 2:** Descriptive statistics of the examinations

Year	Phase	Students	Alpha cronbach	Difficulty Index Mean (SD)	Discrimination Mean (SD)	Flawed items N (%)
		N		0.62 (0.21)	0.34 (0.16)	446 (55.8)
2008	1	217	0.883	0.57 (0.19)	0.33 (0.14)	71
2008	2	123	0.864	0.63 (0.20)	0.32 (0.17)	59
2009	1	208	0.877	0.66 (0.20)	0.34 (0.14)	59
2009	2	113	0.892	0.65 (0.21)	0.37 (0.16)	56
2010	1	192	0.859	0.65 (0.21)	0.30 (0.14)	60
2010	2	116	0.868	0.59 (0.21)	0.32 (0.15)	54
2011	1	243	0.890	0.62 (0.22)	0.37 (0.17)	45
2011	2	48	0.897	0.59 (0.20)	0.37 (0.19)	42

The prevalence of standard questions was 45.8 % (Table 3). The flaw areas with higher prevalence were *Writing the Stem* (19.4 %) and *Writing the Choices* (21.5 %). Additional file 1 shows the prevalence by rule. The difficulty index of the items when they were grouped by flaw areas ranged from 0.59 in *Writing the Stem* category to 0.65 in the Standard group. The discrimination index ranged from 0.31 in *Writing the Stem* category to 0.36 in the Standard and *Content Concerns* categories (Table 3). As sensitivity analysis we estimate the effect of the *Content Concerns* without rule 4 (“Keep the content of each item independent from content of other items on the test”) because is the only rule that by default will increase the discrimination index.

**Table 3:** Psychometric indices by flaw areas with and without rule 4 (“Keep the content of each item independent from content of other items on the test”)

Flaw areas	Number of items N (%)	Difficulty Index Mean (SD)	Discrimination Mean (SD)
Standard^a^	354 (45.8)	0.65 (0.21)	0.36 (0.17)
Content concerns^b^	56 (7.2)	0.63 (0.18)	0.36 (0.16)
Content concerns without rule 4^b^	12 (1.6)	0.61 (0.22)	0.25 (0.15)
Style concerns^b^	47 (6.1)	0.62 (0.20)	0.35 (0.14)
Writing the stem^b^	150 (19.4)	0.59 (0.19)	0.31 (0.14)
Writing the choices^b^	166 (21.5)	0.60 (0.21)	0.33 (0.15)

^a^3/4 reviewers considered that the item had no IWF

^b^3/4 reviewers considered that at least one item had IWF from this category

When the data were analyzed without taking into account rule 4 there was a decrease in the discrimination of the *Content Concerns* area. The difficulty index of the same area suffered no change (Table 3).

In total, 55.8 % of all the items held at least one IWF (Table 4). The percentage of flawed items varied from 45.9 % in the abdomen area to 90.5 % in the imagiology area. The percentage of flawed items was statistically significant (p < 0.001). The items difficulty index was different when the anatomical site was analyzed (p = 0.001). The difficulty index ranged from 0.66 in the Pelvis and Perineum area to 0.52 in the Imagiology area. The discrimination index was also influenced by the anatomical site (p < 0.001). This index was highest in the upper limb area (0.39) and was lowest in the thorax area (0.29).

**Table 4:** Percentage of flaws, difficulty and discrimination indices by content area

	Total items	Flawed N (%)	Difficulty Index		Discrimination Index	
			Mean	SD	Mean	SD
Total	800	446 (55.8)				
Content area						
Abdomen	148	68 (45.9)	0.65	0.19	0.32	0.17
Pelvis and perineum	142	86 (60.6)	0.66	0.20	0.36	0.17
Upper limb	71	34 (47.9)	0.63	0.20	0.39	0.14
Lower limb	70	37 (52.9)	0.59	0.21	0.38	0.17
Neck	123	65 (52.8)	0.57	0.21	0.34	0.16
Thorax	139	90 (64.7)	0.63	0.21	0.29	0.14
Head	86	47 (54.7)	0.60	0.21	0.36	0.15
Imagiology	21	19 (90.5)	0.52	0.21	0.32	0.10
*p* value		<0.001	0.001		<0.001	

**Fig. 2:**
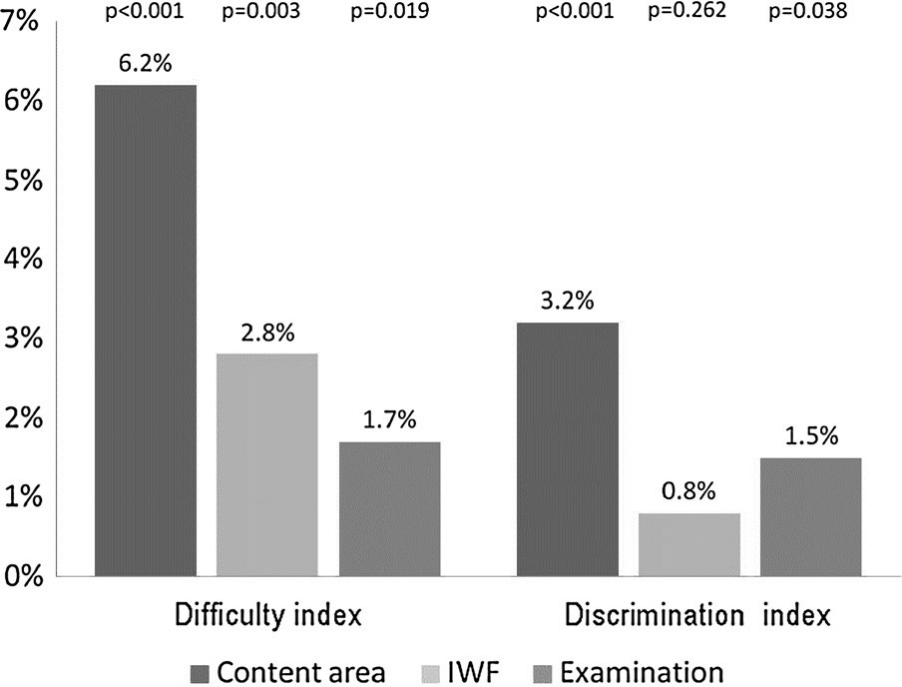
Effect of content area and the presence of item writing flaws adjusted for the examination

The item’s anatomical site explained 6.2 and 3.2 % of the difficulty and discrimination indices, respectively (Fig. 2). These results were statistically significant (p < 0.001 and p < 0.001, respectively). The IWF explained 2.8 and 0.8 % of the difficulty and discrimination indices, respectively (Fig. 2). Only the effect on the difficulty index was statistically significant (p = 0.003). The examination explained 1.7 and 1.5 % of the difficulty and discrimination indices, respectively (Fig. 2). These results are statistically significant (p = 0.019 and p = 0.038, respectively).

## Discussion

The prevalence of flawed items in our study ranged from 42 to 71 % in the different examinations, with a total of 55.8 % of flawed items in all the examinations. This high proportion of the flawed items was similar to other studies, where around half of the analyzed items considered flawed items [7–9]. This result showed the lack of preparation and time invested by teachers in MCQ construction to assess students’ performance [7]. The most prevalent types of flaws were on *Writing the Stem* (19.4 %) and *Writing the Choices* (21.5 %). In the first type rule 17 (“Word the stem positively, avoid negatives such as NOT or EXCEPT”) was the more prevalent (94 %) while in second type rules 22 (11.4 %, “Keep choices independent; choices should not be overlapping”), 24 (51.8 %, “Keep the length of choices about equal”) and 25 (22.9 %, “None-of-the-above should be used carefully”) were the most prevalent (Additional file 1).

The impact of the IWF was heterogeneous, the *Writing the Stem* and *Writing the Choices* categories had a negative impact (higher difficulty and lower discrimination) while the other categories did not have any impact. This suggests that medical teachers should focus mainly on the rules that belong to *Writing the stem* and *Writing the choices* flaws groups. Both had a similar effect, decreasing the difficulty and the discrimination indices of the items. These effects were mainly explained by rule 17 and rule 25 (Additional file 1). Rules about the *Style Concerns* had no effect on the psychometric characteristics (Additional file 2).

Rules about *Content Concerns* showed an effect only in the items discrimination index, decreasing the discrimination, when rule 4 (“Keep the content of each item independent from content of other items on the test”) was discarded. This happens because When rule 4 was violated the discrimination increased as expected, because when a student had a correct answer in one of the dependent items he should have a higher probability of correctly answered the other dependent items and similar effect if the student had a wrong answer.

The discrimination indices can increase from 0.25 (violating the content concerns rules) to 0.36 that according to the medical educators is going from fair to good item discrimination. The removal of this type construct-irrelevant variance will allows to obtain a more valid assessment.

Previous studies showed that violating the IWF rules had a negative impact in the psychometric indices [15]. However, our study showed that the impact was different according to the IWF area.

There were significant differences between anatomical sites and the presence of flawed items. The most explicit difference was in the Imagiology area, with a proportion of flawed items of 90.5 %. The two main IWF present in this category were the presence of negative words in the items stem of and the differences in the options length.

There were significant differences between anatomical sites and psychometric indices. Two possible reasons can be accounted to explain this effect: the specific area of the items contributed to a shift in the psychometric indices (for example, due to a lack of students’ preparation) or the presence of IWF concentrated in certain anatomical site, indirectly altering their psychometric characteristics. For example, Thorax and Imagiology contained the highest proportion of flawed items from all the anatomical sites and, at the same time, were the categories with the lowest discrimination and the lowest difficulty indices, respectively. Another explanation could be that for almost all anatomical sites there was a similar items proportion in the examinations and number of hours that were lectured about that topic. However, when observing one particular area, Neck, about 9 % of the lectures during the course were about the neck area and in the examination, 15 % of the MCQ referred to this content. This discrepancy in the neck area may have implied a lower value in the difficulty index. This discrepancy reflected the lack of existence of an examination blueprint to guide the construction of the examination. This error represents one of the major validity threats caused by the absence of an examination blueprint, “construct under-representation”, in which the number of questions of a specific area is not proportionally represented in an examination when compared to the curriculum of the course [16]. This threat will result in an unbalanced examination, which might affect the proper student assessment.

These last facts are reflected in our main findings. Our study showed that the effect of anatomical site on the item index remained independently of the IWF, showing that differences between anatomic regions were not explained by them. Others explanations like the number of items matching proportionately with the amount of time spent lecturing on them or possible quality of teaching (materials, lectures, etc.) or even cognitive load of student’s anatomy content vary from region to region.

The generalizability of these findings was limited by the fact that it only evaluated the effect in a course of Clinical Anatomy, however, the students, examinations and examination items were not so different from their equivalents in other areas. So it was possible to generalize these findings and apply them to other pre-clinical medical areas present in the medical course to assure a correct and more precise evaluation of medical students. The anatomical sites and the presence of the IWF only explained a small part of the psychometric characteristics of MCQ. Other possible determinants of the characteristics of the items are the cognitive level of the items should be studied.

## Conclusions

It is of extreme importance to eliminate or, at least, diminish the proportion of the IWF in MCQ present in examinations, to ensure the best possible reliability and validity assessment of the examinees. The IWF’s categories “content concerns”, “writing stem” and “writing the options” have negative impact on the psychometric quality of the test. However, it is also important to take into account the anatomical sites of the same items, because they affect in greater extent the psychometric parameters of the questions independently of the IWF. This study discarded as the main explanation from differences between anatomic regions in the psychometric quality of the test the IWF. Future work should focus on understand for example the cognitive load by anatomic region in order to assess if this is the main explanation for these differences between anatomic regions. When constructing MCQ, the focus should be in the topics/areas of the items and only then in the presence of IWF.

## Additional files

10.1186/s13104-016-2202-4 Item-writing flaws by group. 31 Item-writing flaws grouped by content concerns, style concerns, writing the stem and writing the choices.
10.1186/s13104-016-2202-4 Data set.

## Abbreviations

MCQ: multiple-choice questions
IWF: item-writing flaws
FMUP: Faculty of Medicine of University of Porto
