# Supplementary material for: Do item-writing flaws reduce examinations psychometric quality?
Source: BMC Res Notes. 2016 Aug 11;9:399. doi: 10.1186/s13104-016-2202-4 (PMC4982015; doi:10.1186/s13104-016-2202-4)
Supplement: Supplementary file 1 — 10.1186/s13104-016-2202-4 Item-writing flaws by group. 31 Item-writing flaws grouped by content concerns, style concerns, writing the stem and writing the choices. [file 13104_2016_2202_MOESM1_ESM.docx]

**Additional file 1**

| **Group of flaws** | **Item-writing flaws** | **N (%)** |
| --- | --- | --- |
| Content Concerns | 1. Every item should reflect specific content and a single specific mental behavior, as called for in test specifications (two-way grid, test blueprint). | 6 (10.7) |
|  | 2. Base each item on important content to learn; avoid trivial content. | 5 (8.9) |
|  | 4. Keep the content of each item independent from content of other items on the test. | 44 (78.6) |
|  | 6. Avoid opinion-based items. | 1 (1.8) |
|  | Total | 56 (100) |
| Style Concerns | 11. Edit and proof items | 8 (17) |
|  | 12. Use correct grammar, punctuation, capitalization, and spelling | 6 (12.8) |
|  | 13. Minimize the amount of reading in each item | 34 (72.3) |
|  | Total | 47 (102.1) |
| Writing the Stem | 15. Include the central idea in the stem instead of the choices | 5 (3.3) |
|  | 16. Avoid window dressing (excessive verbiage) | 4 (2.7) |
|  | 17. Word the stem positively, avoid negatives such as NOT or EXCEPT. If negative words are used, use the word cautiously and always ensure that the word appears capitalized and boldface | 141 (94) |
|  | Total | 150 (100) |
| Writing the Choices | 19. Make sure that only one of these choices is the right answer | 2 (1.2) |
|  | 22. Keep choices independent; choices should not be overlapping | 19 (11.4) |
|  | 24. Keep the length of choices about equal | 86 (51.8) |
|  | 25. *None-of-the-above* should be used carefully | 38 (22.9) |
|  | 26. Avoid *All-of-the-above* | 13 (7.8) |
|  | 27. Phrase choices positively; avoid negatives such as NOT | 6 (3.6) |
|  | 28. Avoid giving clues to the right answer | 8 (4.8) |
|  | 29. Make all distractors plausible | 1 (0.6) |
|  | 31. Use humor if it is compatible with the teacher and the learning environment | 2 (1.2) |
|  | Total | 166 (105.3) |
